# *In Vivo* Analysis of the Notch Receptor S1 Cleavage

**DOI:** 10.1371/journal.pone.0006728

**Published:** 2009-08-24

**Authors:** Robert J. Lake, Lisa M. Grimm, Alexey Veraksa, Andrew Banos, Spyros Artavanis-Tsakonas

**Affiliations:** 1 Department of Cell Biology, Harvard Medical School, Boston, Massachusetts, United States of America; 2 Biology Department, University of Massachusetts Boston, Boston, Massachusetts, United States of America; 3 Collège de France, Paris, France; Universidade Federal do Rio de Janeiro (UFRJ), Instituto de Biofísica da UFRJ, Brazil

## Abstract

A ligand-independent cleavage (S1) in the extracellular domain of the mammalian Notch receptor results in what is considered to be the canonical heterodimeric form of Notch on the cell surface. The *in vivo* consequences and significance of this cleavage on *Drosophila* Notch signaling remain unclear and contradictory. We determined the cleavage site in *Drosophila* and examined its *in vivo* function by a transgenic analysis of receptors that cannot be cleaved. Our results demonstrate a correlation between loss of cleavage and loss of *in vivo* function of the Notch receptor, supporting the notion that S1 cleavage is an *in vivo* mechanism of Notch signal control.

## Introduction

Intercellular communication is required for the proper specification of cell fates in metazoans. Notch signaling defines a conserved, pleiotropic cell-interaction pathway that controls cell fates and consequently many differentiation, proliferation and apoptotic events throughout development [1]–[3]. The central element of this pathway is the transmembrane Notch receptor, which triggers signaling through interaction with membrane-bound ligands expressed on adjacent cells.

Multiple studies focusing on both the *Drosophila* and mammalian Notch receptors have led to a model for Notch signaling that involves ligand-dependent cleavages of both the extracellular and intracellular domains of the receptor at the plasma membrane. A series of cleavages eventually leads to the release of the intracellular domain, which carries nuclear localization signals [4], from the cell surface followed by its translocation to the nucleus where it participates directly in transcriptional events [5]–[8]. Cell culture experiments suggested that upon ligand stimulation, an extracellular cleavage close to the membrane facilitates a presenilin complex-dependent cleavage that releases the intracellular domain from the cell surface [5], [9]–[11]. Biochemical evidence from mammalian studies has also revealed the existence of a ligand-independent cleavage (S1 cleavage) in the extracellular domain that is responsible for maturation of the protein [12]. This cleavage was shown to depend on the furin protease, a member of the proprotein convertase family of proteases [13]. S1 cleavage apparently occurs in the *trans*-Golgi apparatus and results in the creation of a heterodimeric form of the Notch receptor [12]. This form of the receptor is composed of a 180 kDa cleavage product (NEC) encompassing entirely extracellular sequences, and a 110–120 kDa product (NTM) that includes a short piece of the extracellular domain, and the entire transmembrane and intracellular domains. Biotinylation experiments demonstrate that the heterodimeric receptor is the dominant form of Notch located on the cell surface, even though traces of the full-length protein can clearly be detected [12]–[14]. The association between the two subunits of the heterodimeric *Drosophila* and mammalian Notch receptors appears to depend upon metal ions, by virtue of the fact that chelating agents release the extracellular portion of the receptor and can, in fact, activate downstream signaling [15].

In spite of these biochemical studies, the *in vivo* functional significance and the generality of S1 cleavage remains unclear, and indeed one study has even questioned its existence in *Drosophila* [16]. We sought to address the functional significance of S1 cleavage in *Drosophila* by examining the *in vivo* and *in vitro* biological activity of receptors that have mutated cleavage sites and are, thus, incapable of being cleaved. Our studies involving the analysis of transgenic flies indicate an *in vivo* correlation between S1 cleavage of the Notch receptor and biological activity, supporting the significance of the S1 cleavage for Notch receptor function [12], [13].

## Materials and Methods

### TAP purifications and mass-spectrometric analysis

The cloning of Notch in the TAP vectors, the TAP purifications and the mass-spectrometric analysis were described in Veraksa et al[17].

### Notch mutant construction

Notch mutant forms were generated using the Stratagene site-directed mutagenesis kit and primers with appropriately altered amino acid codons. For the F1and F2 mutations, amino acids in the sequences RKNK and RLKK, beginning at amino acids 1667 and 1637 were mutated to alanines, respectively. The mutations were incorporated into wild-type full-length *Drosophila* Notch (described in [18]) in both the pMT [19] and pUAST [20] vectors.

### Transient transfections

Transient transfections were performed in 6-well plates using 2 µg of DNA and Effectene reagent (Qiagen). Approximately 24 hours after transfection, protein expression was induced by overnight treatment of the cells with 0.35 mM CuSO_4_.

### Western blotting

Following treatment of cells with 0.35 mM CuSO_4_, cells were washed once in 1xPBS and cell pellets were lysed in a detergent-based buffer composed of 50 mM Tris (pH 7.4), 1.0% NP-40, 0.25% sodium deoxycholate, 150 mM NaCl. The lysis buffer was supplemented with the Complete Protease Inhibitor Cocktail, EDTA-free (Roche). Western blot analysis with the anti-Notch C17.9C6 antibody (dilution 1∶6000) was performed according to standard protocol.

### Biotinylation of S2 cells

S2 cells were seeded onto 10 cm tissue culture dishes treated with concanavilin A.

Dishes were incubated in 0.5 mg/ml concanavilin A for 30 minutes and were then washed 3 times with 1 x PBS prior to seeding of cells. Cells were transfected with 6 µg of DNA/plate using Effectene reagent (Qiagen). The following day, cells were treated with 0.35 mM CuSO_4_ and after 16 hours of treatment, cells were biotinylated. All of the following steps were performed on ice. S2 cells were washed 3 times in 1 x PBS and then cells were incubated in 2 mls of biotin (1.0 mg/ml in 1 x PBS pH 8.0) for 15 minutes on a rocking platform. The biotinylation step was repeated. Following biotinylation, 2 mls of 1 x PBS pH 8.0+100 mM glycine were added to quench the reaction. Quenching occurred on a rocking platform for 15 minutes. The quenching reaction was also repeated. Cells were then washed 3 times in 1x PBS pH 8.0 and lysed in RIPA buffer supplemented with the Complete Protease Inhibitor Cocktail, EDTA-free (Roche).

### Rescue of Notch null embryos

For assessing the rescuing ability of the F1 and F2 mutant Notch proteins, *da-GAL4* males [21] were crossed to the females of the following genotypes: *N^54l9^/FM7c ftz-lacZ*, *N^54l9^ UAS-N-WT/FM7c ftz-lacZ*, *N^54l9^ UAS-N-F1/FM7c ftz-lacZ* and *N^54l9^ UAS-N-F2/FM7c ftz-lacZ* to obtain mutant *Notch* embryos and mutant embryos rescued with wild-type Notch, F1 and F2, respectively. Embryos were first scored for the absence of *FM7c ftz-lacZ*. Of those, 50% were wild-type and therefore female, and the remaining 50%, having *N^54l9^*, showed neurogenic phenotypes to varying degrees, depending upon the transgene present. The neurogenic phenotypes were scored by carefully examining neurons of the peripheral nervous system. These neurons were central to diagnosing the extent of neurogenic rescue. A minimum of 25 rescued embryos was examined for each genotype. Thus, *N^54l9^* mutant embryos were unambiguously identified by their neurogenic phenotype, which was not completely rescued even by wild-type Notch, and additionally confirmed by the absence of beta-galactosidase staining.

Embryos were fixed in 4% formaldehyde (Polysciences) in Embryo Fix Buffer (10 mM K_2_HPO_4_/KH_2_PO_4_ pH 6.8, 45 mM KCl, 25 mM NaCl, 2 mM MgCl_2_) as an emulsion with heptane on a shaking platform, devitellinized and stored in 100% methanol. Embryos were permeabilized with Tri-PBS (phosphate buffered saline with 0.2% Triton X-100) and incubated with rat anti-ELAV antibody 7E8A10 (Developmental Studies Hybridoma Bank, 1∶100) and rabbit anti-beta-galactosidase (Molecular Probes, 1∶1000) overnight at 4°C in Tri-PBS with 0.1% BSA and 8% goat serum. After multiple washes, embryos were incubated with FITC-conjugated goat anti-rabbit and Cy3-conjugated goat anti-rat antibodies (Jackson Immunoresearch) at room temperature. Embryos were mounted in Fluoroguard (BioRad), and the signal was detected using Zeiss LSM510 confocal imaging system.

### Fly Strains

All transgenic flies were generated in a *w^1118^* background. Germline transformation was performed by the MGH core facility using standard methods.

## Results

### Analysis of the NTM fragment

**Figure 1:**
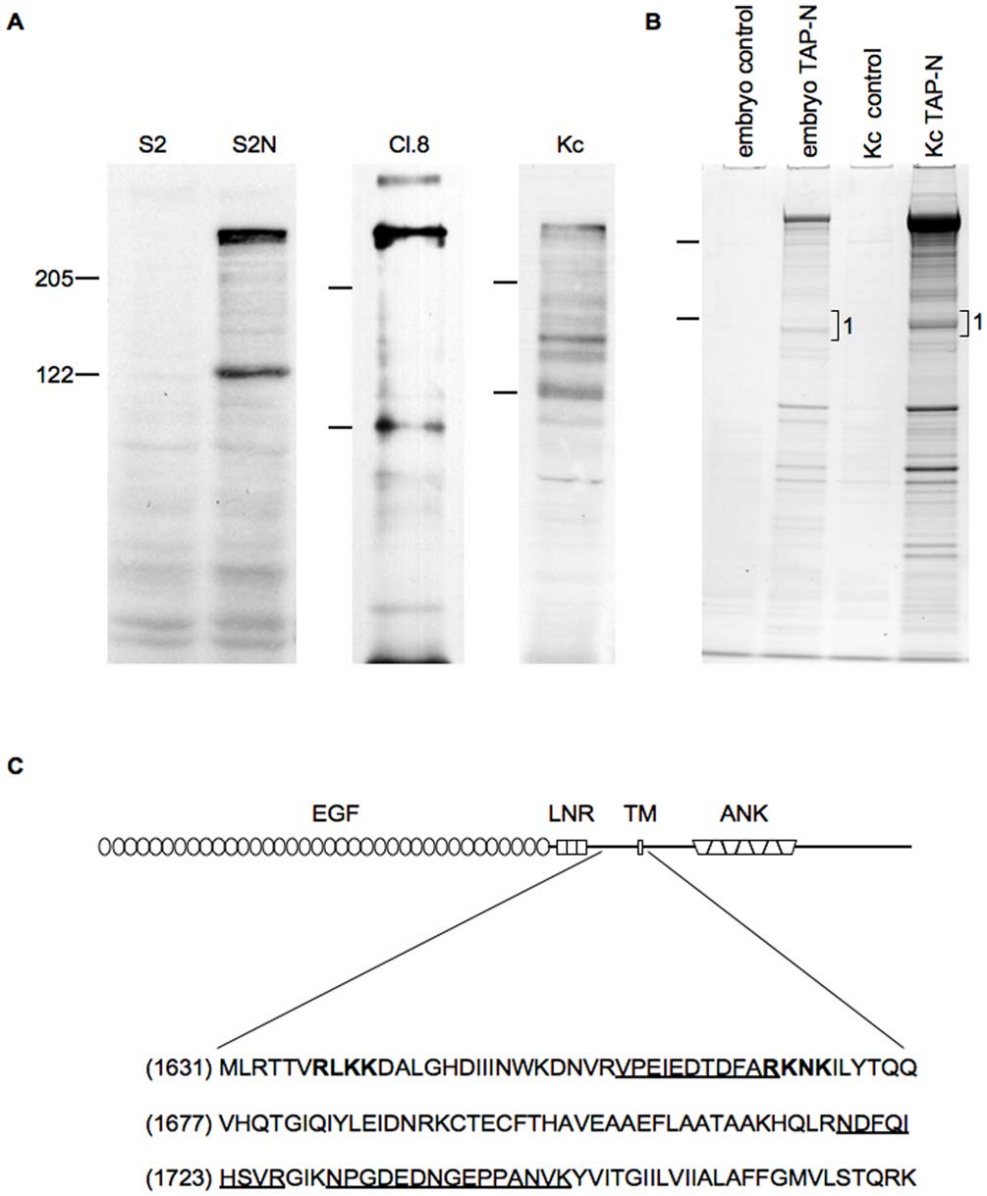
Analysis of the N-terminus of the NTM fragment. (A) Whole cell extracts from the *Drosophila* cell lines S2, S2-Notch (S2N), Clone 8 (Cl.8), and Kc167 (Kc) were analyzed by Western blot using C17.9C6 antibody, an antibody specific for the intracellular domain of Notch. (B) Notch polypeptides from Kc cells and embryos stably expressing a full length Notch construct containing a TAP tag at the C-terminus of its intracellular domain were purified, resolved by SDS PAGE electrophoresis and stained with Coomassie Blue. Polypeptides resolving in the region marked 1 were excised from the gel and submitted for sequencing by mass spectrometry. (C) Schematic representation of the full-length Notch protein and the sequence surrounding the most N-terminal, trypsin-generated peptides (underlined) originating from region 1 and residing in the extracellular domain. Amino acids shown in bold are putative S1 cleavage sites mutated in this study. EGF, EGF repeats; LNR, Lin-12 Notch repeats; TM, transmembrane domain; ANK, ankyrin repeats.

Western blot analyses of the Notch receptor in different tissues, developmental stages and species have long established the existence of multiple cleavage products (e.g. [18]) The pattern revealed by these analyses is variable and can differ both qualitatively and quantitatively, depending on the protein source. However, Western blot analyses of protein extracts from *Drosophila* cells, expressing either a transgene encoding a full-length receptor (S2 cells) or cells endogenously expressing Notch (Cl.8 and Kc167), reveal a consistent and prominent pattern of Notch proteolytic fragments. The 9C6 antibody, specific for the intracellular domain of Notch, detects a variety of polypeptides including full-length Notch and the prominent 120 kDa NTM fragment (Figure 1A). This fragment appears even in the absence of ligand, as demonstrated by its presence in S2N cells which do not express Delta or Serrate [18], indicating that the 120 kDa NTM polypeptide is the product of ligand-independent cleavage.

**Figure 2:**
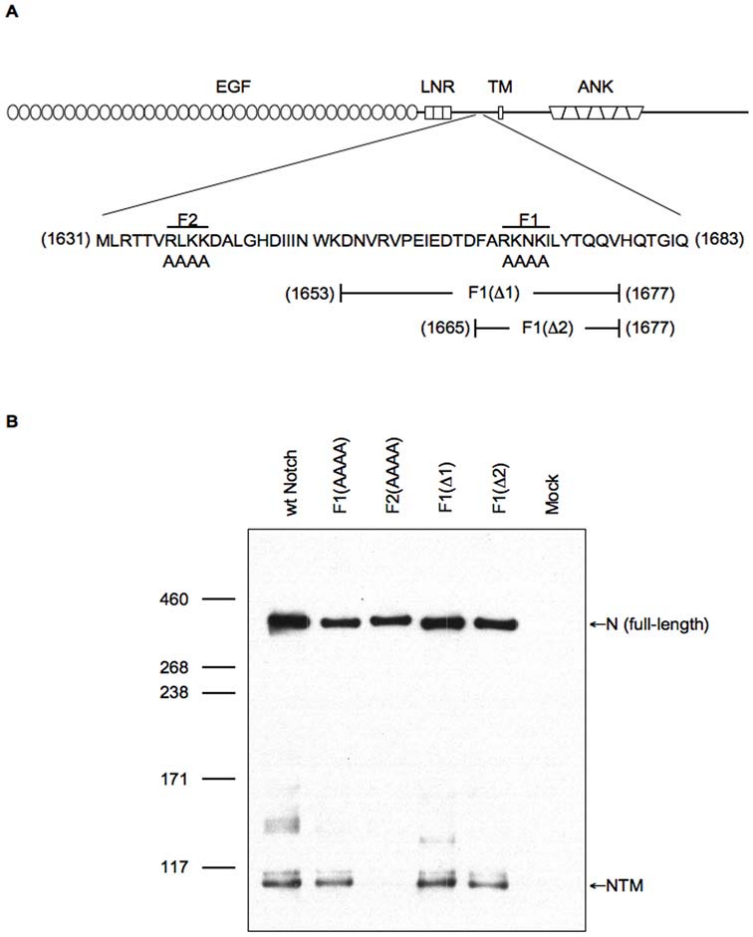
Capacity of Notch, mutated at two putative cleavage sites, to generate the NTM fragment. (A) Schematic diagram illustrating the Notch protein and the amino acid sequence encompassing the two predicted S1 cleavage sites, RLKK (F2) and RKNK (F1). Mutant Notch proteins containing alanine substitutions of the F2 or F1 site, and the two F1-site deletions (F1(Δ1) and F1(Δ2)) are as indicated. (B) Western blot analysis of lysates prepared from S2 cells transiently expressing wild-type Notch (wt-Notch) or the mutant Notch proteins. Untransfected cells are shown as a control (Mock). The Western blot was probed with the C17.9C6 antibody.

In an effort to precisely define the N-terminus of the *Drosophila* NTM, we attempted to directly sequence this protein fragment but were unable to obtain reliable sequence data, possibly due to blockage of the N-terminus. We thus employed mass-spectrometric analysis to estimate the position of the N terminus of NTM. A full-length Notch construct containing a tandem affinity purification (TAP) tag at the C-terminus was generated and stably expressed in both *Drosophila* embryos and Kc167 cells. The TAP tag allows for two-step protein purification [17], [22], [23]. TAP-tagged Notch proteins were purified and resolved by SDS-PAGE, and the region in the 120 kDa range (Figure 1, region 1) was analyzed by mass spectrometry (LC-MS/MS) (Figure 1B). The most N-terminal amino acid from the Notch polypeptides in region 1 corresponded to residue 1658 of the extracellular domain (Figure 1B,C), confirming that the Notch polypeptides in the 120 kDa molecular weight range are membrane bound. Given the study by Logeat et al. [13] that linked S1 cleavage in mammalian cells to the existence of a functional Furin cleavage site, it is noteworthy that the most N-terminal Notch fragment in region 1 mapped close to, but not beyond, one of two putative sites as defined by close homology to the Furin cleavage site consensus (see below). This site, F2 (RLKK), is located at amino acids 1637 through 1640 (Figure 2). We note that full-length Notch was a predominant isoform of Notch in the TAP experiments.

### Mutation of the F2 site results in loss of NTM

Our approach to assess the functional significance of S1 cleavage was to monitor the activity of Notch proteins that have been mutated such that cleavage is impaired. Using the results obtained from the mass-spectrometric analysis as a basis to locate putative Furin cleavage sites, we identified two regions containing basic amino acids that conform to a Furin-target consensus sequence and, if cleaved, would produce a fragment of approximately 120 kDa: RLKK (amino acids 1637–1640) and RKNK (amino acids 1667–1670). We note, however, that neither of these sites form a perfect match to the Furin consensus sequence (Molloy et al., 1992; Walker et al., 1994). These amino acids were mutated to alanines to generate F2 Notch and F1 Notch, respectively (Figure 2A). We also generated two deletion constructs, F1(Δ1) and F1(Δ2), that remove the F1 cleavage site (RKNK) and surrounding residues. Western blot analysis of S2 cells transiently expressing these constructs demonstrated a large reduction in levels of the NTM fragment in cells expressing the F2 protein but not in cells expressing Notch proteins mutant for the F1 site (Figure 2B). This result suggests that the RLKK sequence and not the RKNK sequence is the site of S1 cleavage, agreeing with the prediction from our mass-spectrometric analysis (Figure 1), and a result also consistent with the observations of Hu et al. [24] involving mutagenesis of the RLKK site.

### Blockage of S1 cleavage results in a mutant receptor

**Figure 3:**
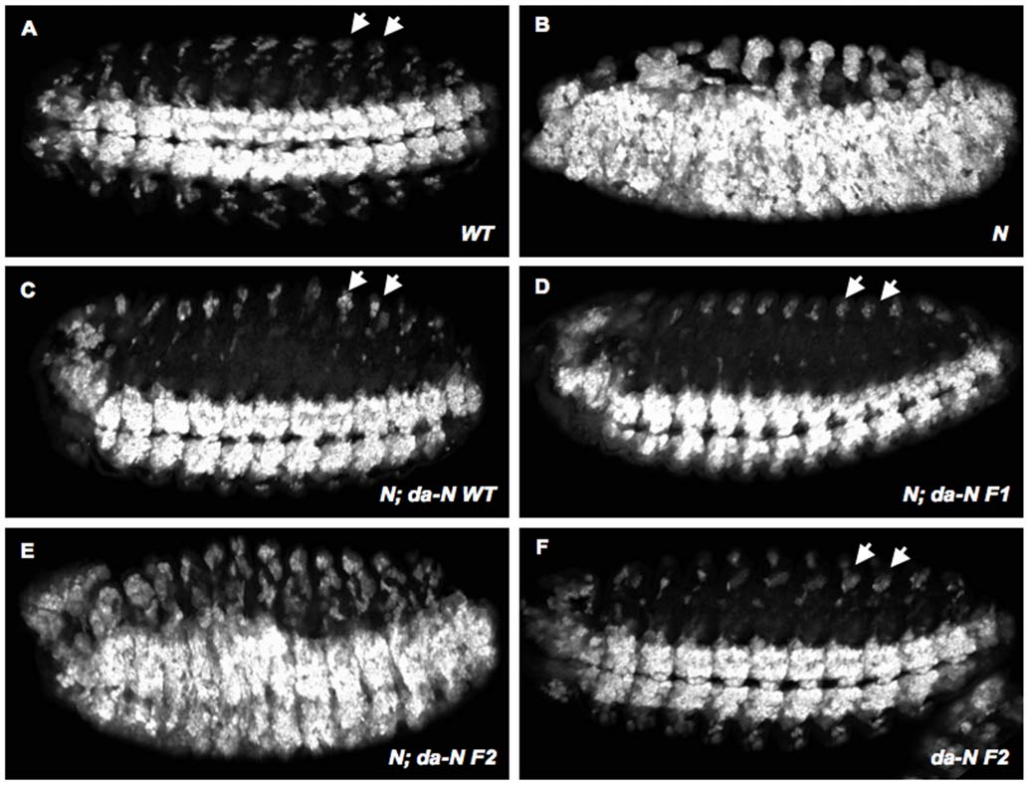
Rescue of the *Notch* neurogenic phenotype by the F1 and F2 mutants. (A–F) Ventro-lateral views of stage 13 embryos stained with anti-ELAV antibody to visualize the extent of development of the nervous system. Anterior is to the left. (A) Wild-type embryos had a normal ventral nerve cord and properly differentiated peripheral nervous system. (B) *N^54l9^* embryos displayed a classic neurogenic phenotype consisting of overproliferated ventral and peripheral nervous system. These defects were largely suppressed by expressing either the wild-type (C) or F1 mutant Notch (D) receptors under the control of the *da-GAL4* driver. In contrast, no significant rescue was observed when F2 mutant Notch was expressed (E). Overexpression of the F2 mutant form in wild-type embryos did not result in nervous system defects (F). Arrows in A, C, D and F point to neurons of the peripheral nervous system, which were used to assess the extent of rescue. A minimum of 25 embryos was examined for each genotype.

**Figure 4:**
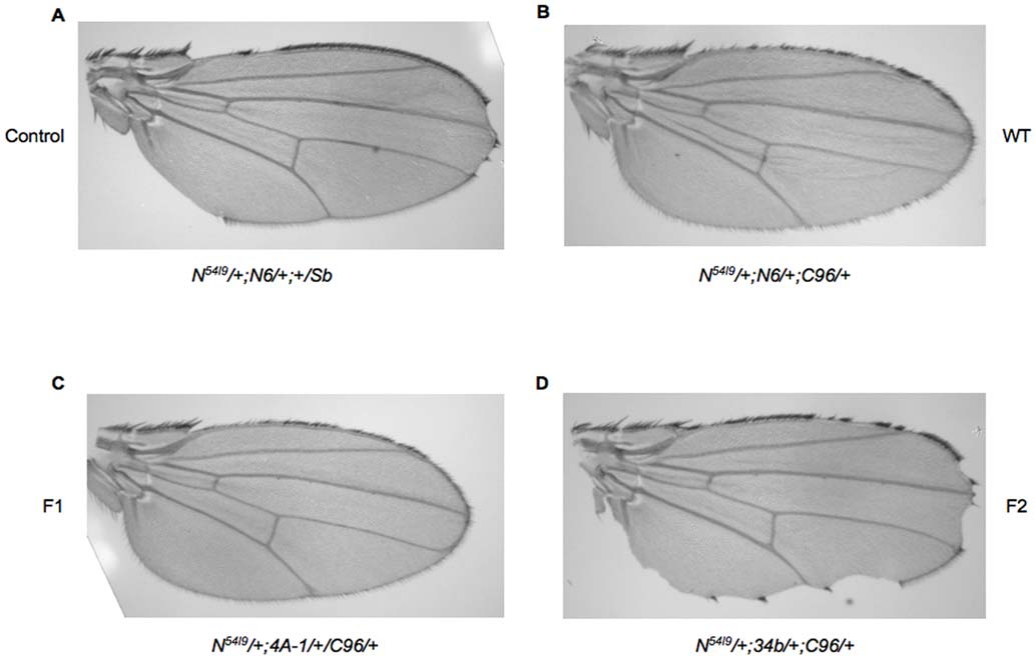
Ability of mutant receptors to rescue Notch loss-of-function wing phenotypes. Transgenic flies overexpressing wild-type Notch (B), F1 Notch (C) or F2 Notch (D) under the control of the wing-specific *C96 Gal4* driver were crossed to *N^54l9^/+*flies (A). Wings from the female progeny of these crosses are shown. A minimum of 50 wings was examined for each genotype. The expressivity of of *N^54L9^* was not variable.

In order to gain insight into the functional significance of S1 cleavage, we compared the ability of wild-type, F1 and F2 Notch receptors to rescue the neurogenic phenotype associated with zygotic loss of *Notch* function. When analyzed with the neuronal marker ELAV, Notch null embryos (*N^54l9^*) display hypertrophy of the central and peripheral nervous systems (Figure 3B) [25]. Under the control of the ubiquitous *da-Gal4* driver [21], wild-type, F1 and F2 Notch transgenes were expressed in *N^54l9^* mutant embryos, and the resulting phenotypes were compared to the neurogenic phenotype displayed by *N^54l9^* embryos. Expression of wild-type Notch or the F1 mutant receptor, which are readily cleaved, significantly rescued the neurogenic phenotype (Figure 3C–3D). The F2 mutant receptor, which displayed impaired cleavage in S2 cells (Figure 2B), did not rescue the neurogenic phenotype (Figure 3E). We also examined the ability of the mutant receptors to rescue the haploinsufficient, notched-wing phenotype resulting from loss of one *Notch* allele (*N^54l9^/+*). Similar to the rescue experiments in Notch null embryos, the wild-type and F1 receptors suppressed the notched-wing phenotype, while the F2 receptor did not (Figure 4). Interestingly, the F2 receptor can enhance the hypomorphic notched-wing phenotype; however, the underlying molecular mechanism causing this enhancement awaits further study. We conclude that the form of Notch with impaired S1 cleavage (F2 Notch), does not behave as wild-type Notch, while the form capable of being cleaved (F1 Notch) has a biological activity that is indistinguishable from the wild-type receptor in these assays.

### Loss of S1 cleavage impairs Notch trafficking

**Figure 5:**
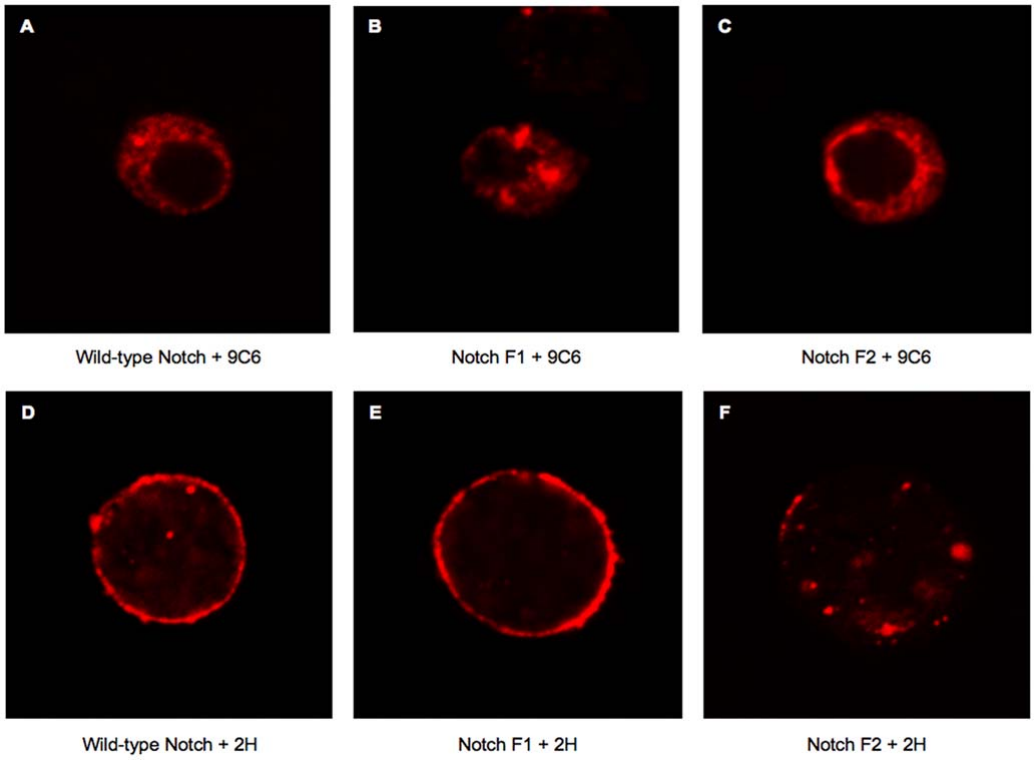
Localization of mutant receptors by immunostaining. S2 cells were transiently transfected with wild-type, F1 or F2 receptors. Cells were immunostained with C17.9C6 antibody after permeabilization with triton X-100 (A,B,C) or were immunostained with C458.2H in the absence of permeabilization (D,E,F). Representative cells from multiple fields are shown.

The original study which defined S1 cleavage was based on the human Notch2 receptor and established that this cleavage is sensitive to Brefeldin A and led to the suggestion that this cleavage is necessary for the receptor to reach the cell surface [12]. This notion was later confirmed and extended by the expression of mammalian Notch receptors containing mutations in predicted Furin cleavage sites [13]. In an effort to characterize further the consequences of interfering with S1 cleavage of *Drosophila* Notch, we examined the subcellular localization of the F2 and F1 mutant Notch proteins and compared their distribution to that of the wild-type receptor. S2 cells transiently expressing wild-type, F1 or F2 receptors were immunostained with antibodies that recognize epitopes in the intracellular (9C6) or extracellular (2H) domains (Figure 5). S2 cells do not express Notch endogenously, and therefore the only proteins contributing to the immunostaining were those introduced through transfection. Staining with the 9C6 antibody revealed the presence of all three proteins in the cytoplasm (Figure 5A–5C). Treating nonpermeabilized cells with the 2H antibody revealed staining around the periphery of cells expressing wild-type and F1 Notch, but staining around the periphery of cells expressing the F2 Notch protein was significantly reduced (Figure 5D–5F). A lack of cell-surface accumulation was also observed when F2 Notch was expressed in wing imaginal discs (data not shown).

**Figure 6:**
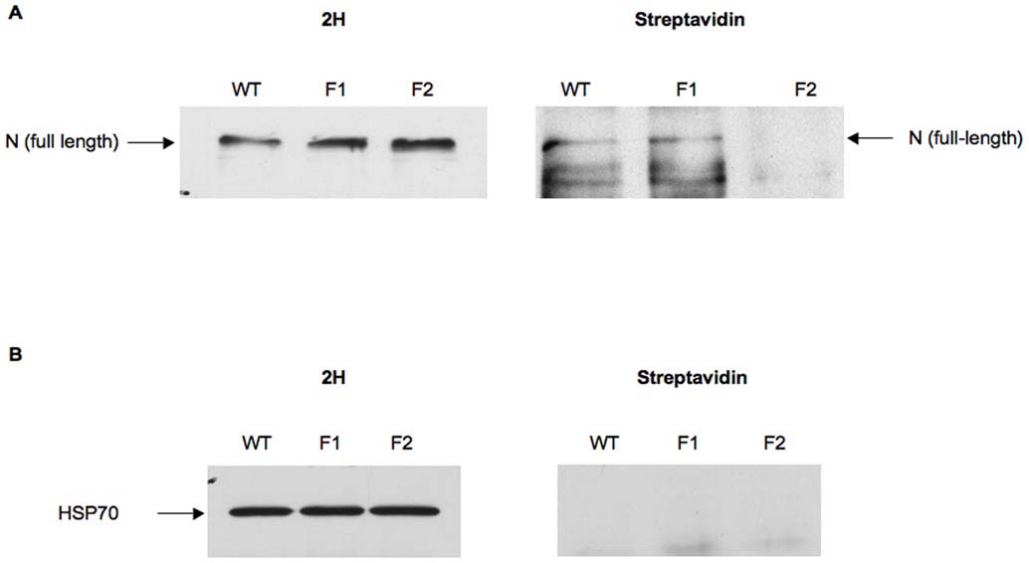
Localization of mutant receptors by biotinylation. S2 cells were transiently transfected with wild-type, F1 or F2 receptors. Cells were biotinylated and immunoprecipitated with the C458.2H antibody and proteins were immunoblotted with either C458.2H (1∶500) (A, left side) or streptavidin-HRP (1∶3000) (A, right side). Biotinylation of the intracellular heat shock 70 protein was monitored as a negative control (B).

In an effort to examine the surface accumulation of the mutant Notch receptors in a more quantitative manner, biotinylation experiments were performed. These studies confirmed our observation that trafficking of the full-length F2 receptor to the cell surface was impaired, as shown by significantly reduced levels of biotinylated F2 Notch (Figure 6A, streptavidin blot), even though the F2 receptor was expressed to relatively high levels as assessed by probing the Western blot with the 2H antibody (Figure 6A, 2H). For technical reasons, the presence of the heterodimeric form of the wild-type Notch receptor at the cell surface could not be confirmed in this biotinylation experiment. Therefore, although a biotinylated fragment of the appropriate size for NEC is observed in the streptavidin blot (data not shown), its absence in the 2H blot prevents confirmation that it is a Notch fragment. In order to assess the integrity of the cell membrane in these experiments and, thus, exclude the possibility of labeling cytoplasmic proteins, the biotinylation status of the intracellular heat shock protein 70 (hsp70) was monitored. Hsp70 was not biotinylated, confirming that the plasma membrane remained intact during the biotinylation process (Figure 6B). These results demonstrate that mutating the RLKK site, which has been shown to prevent S1 cleavage, reduces the accumulation of Notch at the cell surface. They also offer an explanation for the absence of F2 Notch activity in *Drosophila*, as its inability to reach the plasma membrane would prevent it from binding ligand on a neighboring cell.

### Reduced levels of Furin do not impact S1 cleavage

**Figure 7:**
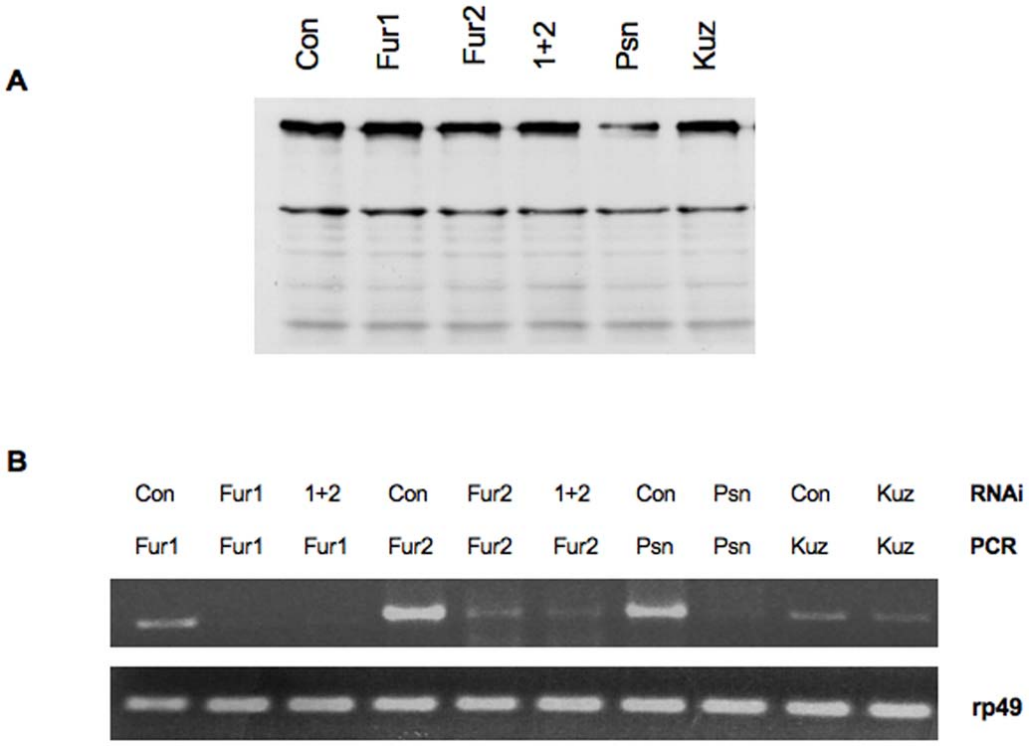
Notch cleavage patterns after treatment with dsRNA. (A) S2 cells were incubated for 4 days with 50 µgs of dsRNA targeting beta-lactamase (Con.), furin 1 (fur 1), furin 2 (fur2), furin 1+2 (1+2), presenilin (psn) or kuzbanian (kuz). After day 3 of dsRNA treatment, cells were transeintly transfected with Notch and lysates were subjected to Western blot analysis using the C17.9C6 antibody. (B) RNA was collected from the cells after completion of the dsRNA and transfection treatments and RT-PCR analysis was performed. RT-PCR using primers to rp49 served as a loading control.

Data obtained from mammalian cell culture studies are consistent with the notion that S1 cleavage depends on Furin activity. We, therefore, attempted to correlate S1 cleavage with the activity of the two annotated *Drosophila* Furins using an RNA interference approach in cultured cells, or by treating cells with the mammalian Furin α1-PDX inhibitor [26]. Reduction in Furin1 and Furin2 activity, either independently or in combination, by RNAi or by treatment with α1-PDX, did not reveal an obvious reduction of S1 cleavage (Figure 7). In addition, we extended these studies to an *in vivo* system by expressing the α1-PDX inhibitor in *Drosophila* wings using different wing drivers. Expression of the Furin inhibitor did not disrupt normal wing development (data not shown). These observations raise the possibility that an enzyme other than Furin may be responsible for S1 cleavage in *Drosophila*. However, the limits of the RNAi approach and the lack of clear, *Drosophila*-specific positive controls prevent us from reaching such a conclusion with certainty.

## Discussion

Proteolytic events are increasingly recognized to play important roles in the function of both the receptor and the ligand in Notch signaling. The model of Notch cleavage that has developed from the accumulated evidence of many different laboratories has Notch being cleaved in its extracellular domain by Furin (S1) as it traffics to the cell surface. Upon ligand interaction, Notch is again cleaved in its extracellular domain by a metalloprotease (S2). This proteolytic event facilitates a final presenilin-dependent cleavage (S3) in the intramembrane region that is critical for nuclear translocation of the intracellular domain and signaling. The functional analysis of these events has been complicated by many factors, given that the critical quantities of the signaling fragments *in vivo* are, as a rule, below detectable levels, and their existence has to be inferred indirectly. It is also possible that the current model excludes other functionally relevant cleavages, as Western blot analyses of the Notch protein invariably reveal a complex cleavage pattern.

It is also important to note that some of the proteases and substrates in the Notch signaling pathway are also implicated in mutual interactions and lack stringent cleavage sites, making a genetic analysis of these processes difficult. For example, Kuzbanian is an ADAM metalloprotease that was shown to be involved in cleavage of both the Notch receptor and its ligands [1], [27]–[30]. Kuzbanian is, itself, thought to be processed and activated by the proprotein convertase Furin (Wang Yale Ph.D. thesis). Presenilin, which is well documented to cleave the Notch receptor, has been shown to cleave the receptor's ligands [30]. It is therefore difficult to interpret the genetic interactions between such elements that display these complex relationships.

Given these difficulties, we reasoned that an *in vivo* functional analysis of S1 cleavage of the Notch receptor was required. A similar, cell culture-based approach was used in an S1 cleavage study of the human Notch1 receptor [13]. This study showed that loss of S1 cleavage was accompanied by a reduction in Notch signaling and transport to the cell surface, consistent with our *in vivo* functional data. The mass-spectrometric analysis we carried out revealing the existence of NTM fragments both in *Drosophila* embryos and in cultured cells, and the functional analysis we performed are not in agreement with the conclusions of a study claiming that S1 cleavage is neither essential for function nor even present in *Drosophila* [8]. In this study, a region of *Drosophila* Notch including and surrounding the RLKK (F2) and RKNK (F1) sites was deleted (termed N^BCLexA^) and found to impair receptor trafficking to the cell surface, which is in agreement with our results for the F2 receptor. However, in the rescue experiments of the neurogenic phenotype in N null embryos, N^BCLexA^ did display activity similar to wild-type Notch, in that it was able to suppress, although incompletely, the neurogenic phenotype, implying that some mutant receptor may actually be functional on the cell surface. In our rescue studies in which we examined two developmental contexts, the wing and the embryo, the F2 mutant receptor did not rescue the loss-of-function phenotypes. In an accompanying manuscript, Gordon et al. show that mutation of the S1 cleavage site of the human Notch 1 receptor interfered with the transport of this protein to the cell surface and its function, consistent with our observations. In contrast, mutation of the Notch 2 S1 cleavage site had no effect on transport or function.

*Drosophila* Notch is closest to the mammalian Notch 1 receptor, but in the absence of structural data we cannot directly compare the *Drosophila* receptor with its mammalian counterpart. Nevertheless, it is noteworthy that the results of Gordon et al regarding Notch 1 are at face value consistent with our observations in *Drosophila*, which establish, *in vivo*, a correlation between Notch cleavage and activity. In the absence of structural data, however, any structure/function approach, such as the one we adopted here, suffers from the possibility that the functional changes we observe in the mutant receptor (F2) are the result of incorrect protein folding rather than inhibition of the S1 cleavage *per se*. This caveat remains even though a similar group of mutations at the F1 site, just a few amino acids away from the F2 site, did not disrupt activity of the Notch protein in vivo. It is noted, however, that structural data obtained from an X-ray crystallographic analysis of a small region of the human Notch 2 extracellular domain suggests that amino acids within the F2 site are important for structural contacts [31].

Given that the quantity of competent receptors on the cell surface is a crucial parameter for the developmental outcome of Notch signals, the existence of processing events such as the S1 cleavage provide cells with an important layer of Notch signal control. Whether this cleavage, however, is used to modulate Notch activity in all cells remains to be determined, but given all available evidence, it seems likely that the functional significance of the S1 cleavage may vary and dependent on the developmental context.
